# Dietary manipulation: a sustainable way to mitigate methane emissions from ruminants

**DOI:** 10.1186/s40781-018-0175-7

**Published:** 2018-06-18

**Authors:** Md Najmul Haque

**Affiliations:** Bangabandhu Sheikh Mujibur Rahman Science and Technology University, Gopalganj, 8100 Bangladesh

**Keywords:** Diet, Starch, Sugar, Fibres, Greenhouse gas

## Abstract

Methane emission from the enteric fermentation of ruminant livestock is a main source of greenhouse gas (GHG) emission and a major concern for global warming. Methane emission is also associated with dietary energy lose; hence, reduce feed efficiency. Due to the negative environmental impacts, methane mitigation has come forward in last few decades. To date numerous efforts were made in order to reduce methane emission from ruminants. No table mitigation approaches are rumen manipulation, alteration of rumen fermentation, modification of rumen microbial biodiversity by different means and rarely by animal manipulations. However, a comprehensive exploration for a sustainable methane mitigation approach is still lacking. Dietary modification is directly linked to changes in the rumen fermentation pattern and types of end products. Studies showed that changing fermentation pattern is one of the most effective ways of methane abatement. Desirable dietary changes provide two fold benefits i.e. improve production and reduce GHG emissions. Therefore, the aim of this review is to discuss biology of methane emission from ruminants and its mitigation through dietary manipulation.

## Background

Livestock contribute to global climate change by emitting GHG either directly (from enteric fermentation and manure management) or indirectly (from feed production and the processing and converting of forest into pasture). The major GHGs from the livestock sector are carbon dioxide (CO_2_), methane (CH_4_) and nitrous oxide (N_2_O) throughout the production process (Fig. 1.1). The CO_2_ that is emitted from livestock is not considered a net contributor to climate change because the animals consume plants that use CO_2_ during photosynthesis (Steinfeld et al., 2006). Consequently, CH_4_ and N_2_O are the most important GHGs from the animal production system and have very high global warming potentials (GWP) of 25 and 298 CO_2_ equivalent (eq), respectively [1]. The first comprehensive analysis of the environmental impact of livestock production [2] reported that approximately 18% of the global anthropogenic GHG is contributed by livestock production. The global anthropogenic GHG emissions from agriculture were 5.1 to 6.1 Gigatonnes CO_2_-eq in 2005, of which livestock shared approximately 9% [3]. Within livestock, ruminant supply chains are the main contributors to the GHG, estimating approximately 80% of the total sector’s emissions [4], while non-ruminants, e.g., pigs and poultry, contribute only approximately 9 and 8%, respectively, to the sector’s emissions [5]. The emissions from beef and milk production represent 35 and 30% of the livestock sector emissions, globally. Buffalos and small ruminant supply chains have a much lower contribution, representing 8.7 and 6.7% of sector emissions, respectively [4]. Another report [5] that stated GHG emissions along livestock supply chains estimated approximately 14.5% of all human-induced emissions. Enteric fermentation and feed production related activities in ruminant production are the primary sources of GHG emissions, representing approximately 39 and 45% of the GHG of the total sector’s emissions. The largest source of GHG emissions from ruminant production, i.e., CH_4_ derive from enteric fermentation, which accounts for approximately 47%, greater than 90% of the total CH_4_ emissions [4]. According to the US Environmental Protection Agency in 2009, CH_4_ emissions from enteric fermentation represented approximately 20% of total CH_4_ emissions from anthropogenic sources [6]. The rate of emission in terms of carbon footprint at the product levels is 2.8, 3.4 and 6.5 kg CO_2_-eq/kg FPCM for milk production from dairy cattle, buffalo and small ruminants, respectively. However, with regard to meat from ruminants, the carbon footprint for beef, buffalo meat and small ruminant meat is 46.2, 53.4 and 23.8 kg CO_2_-eq/kg meat, respectively [4]. According to the values that were projected by EPA [7], the direct non-CO_2_ emissions from livestock would be approximately 7.3 to 7.5% of the global GHG emissions between 2010 and 2020, respectively. Ruminant production faces difficult challenges and must reduce GHG emission while responding to the significant demand of livestock products (projected + 70% by 2050 for a world-projected population of 9.6 billion) [5]. The global food demand will also increase with the rapidly increasing global population. Consequently, the demand for animal products will also increase. Therefore, the environmental impact per unit of animal products will obviously be increased. Thus, the sector will be vulnerable in terms of environmental sustainability. Therefore, sustainable and immediate mitigation strategies are in high demand. This review will focus on CH_4_ mitigation from ruminants through dietary manipulation.

**Fig. 1:**
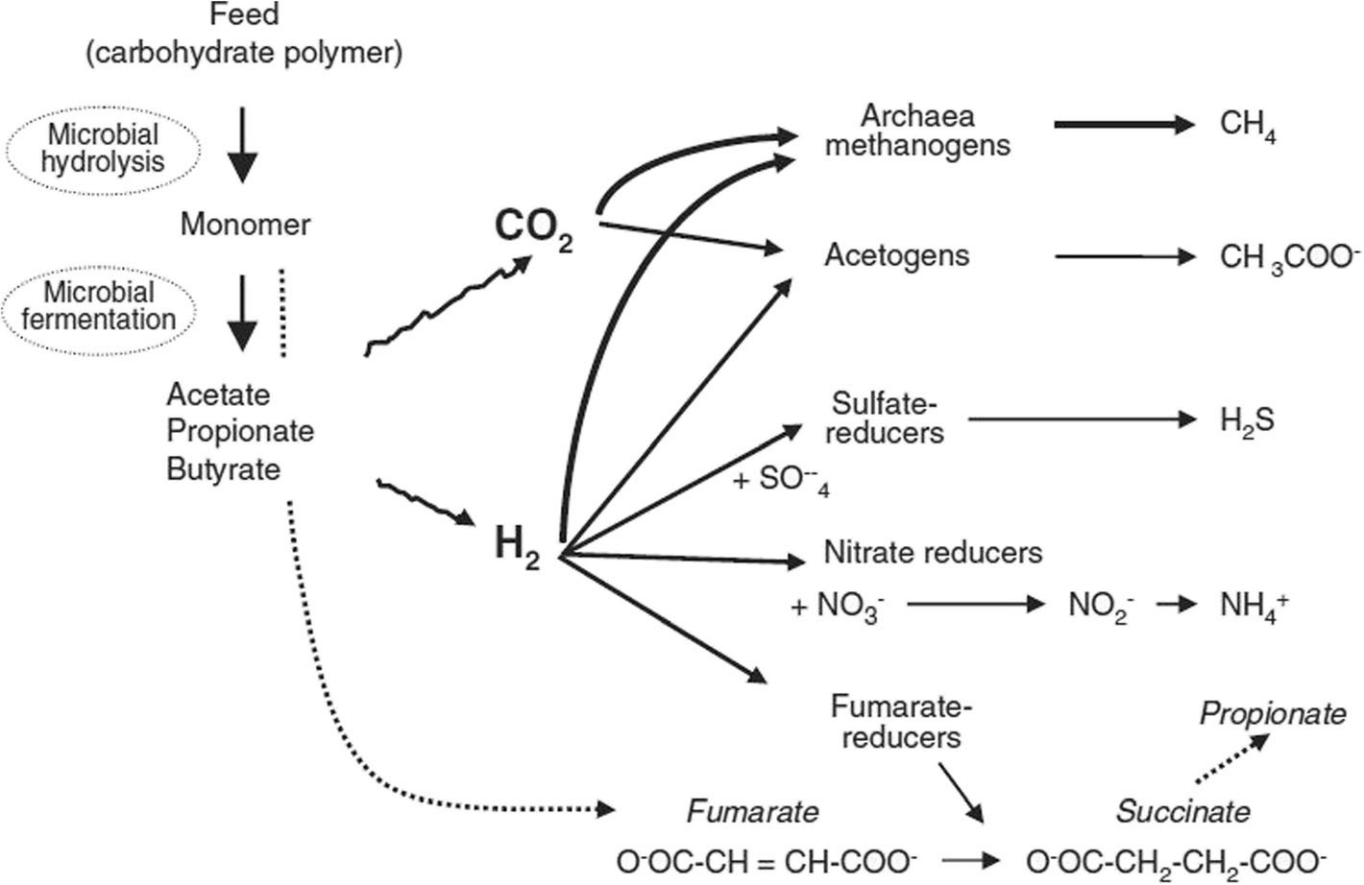
Schematic microbial fermentation and the H_2_ reduction pathway in the rumen [15]

### Methanogenesis and methane production in the rumen

Methanogenesis is a process of CH_4_ production in the rumen where H_2_ reduced the CO_2_ with the help of methanogenic archaea. This is a dynamic process, in which methanogens strongly influence the metabolism of fermentative and acetogenic bacteria via interspecies hydrogen transfer [8]. The carbohydrate fraction of the feed constitutes structural plant fibre that has been degraded by a consortium of rumen microbes under anaerobic conditions with the production of volatile fatty acids (VFA), CO_2_ and H_2_ (summarised in Table). During fermentation, hydrogen (H_2_) is released into the rumen via the re-oxidation of the reduced cofactors (NADH, NADPH and FADH). The produced H_2_ and CO_2_ are the major substrates that are used by methanogens, which is considered being the predominant pathway of CH_4_ production in the rumen [9]. Methane production from H_2_ and CO_2_ reduces the partial pressure of H_2_, thereby favouring continued fermentation [9]. Without the removal of H_2_, the further re-oxidation of reduced cofactors (NADH, NADPH and FADH) would be inhibited by the accumulation of H_2_, consequently inhibiting the production of VFA (Table 1) [10].

**Table 1:** Volatile fatty acids production (VFA) and reductive process in the rumen adopted from [95, 96]

Substrate		Products	∆G (KJ)^1^	Reactions
VFA production				
C_6_H_12_O_6_ + 2H_2_O	→	2 C_2_H_4_O_2_ + 2 CO_2_ + 8H^+^		Acetate production
C_6_H_12_O_6_ + 4H^+^	→	2 C_3_H_6_O_3_ + 2 H_2_O		Propionate production
C_6_H_12_O_6_	→	C_4_H_8_O_4_ + 2 CO_2_ + 4H^+^		Butyrate production
Reductive process				
CO_2_ + 4H_2_	→	CH_4_ + 2 H_2_O	− 67.4	Methane production
2 CO_2_ + 4H_2_	→	C_2_H_4_O_2_ + 2 H_2_O	−8.8	Reductive acetogenesis
SO_4_^2−^ + 4H_2_ + H^+^	→	HS^−^ + 4 H_2_O	− 84.4	Sulfate reduction
NO_3_^−^ + 4H_2_ + 2H^+^	→	NH_4_ + 3 H_2_O	− 371	Nitrate reduction

^1^under following rumen conditions: H_2_ = 162 pa; pH = 6.5; [H_2_O] = 50 M; [succinate^2−^] = 4 × 10^− 6^ M; [malate^2−^] = [β-hydroxybutyryl-CoA] = [butyryl-CoA] = 10^− 6^ M; [acetate^−^] = 70 mM; [propionate^−^] = 25 mM; [butyrate^−^] = 15 mM; [lactate^−^] = 1 mM; [NH_4_^+^] = 11 mM (20 mg/dL); [HS^−^] = 0.14 mM. ∆G = free energy change indicates how energetically favourable it is i.e. the higher ∆G, the more energy utilization and negative ∆G indicates the energy release

In addition, the functional group of methanogens also uses formate, acetate, methanol, methylamines (mono-, di- and trimethylamine) and alcohol [9] as presented in Fig. 1. Formate is used by many of hydrogenotrophic rumen methanogens as an alternative to H_2_ [11], accounting for up to 18% of the total CH_4_ production in the rumen [12]. Acetate is highly available in the rumen environment, but acetoclastic methanogenesis bears very limited importance in the rumen system [13] because the acetate-utilising methanogen *Methanosarcinales* has a very low growth rate and is consequently flushed from the ruminants digestive system [14]. Furthermore, acetogens have a lower affinity to H_2_ [15]. Other substrates, including methylamine and methanol, have been investigated for CH_4_ production in the rumen. The methyl group is rapid converted by the rumen microorganisms to trimethylamine via di- and monomethylamine and is possibly used for CH_4_ production [16]. However, only *Methylotrophic* methanogens within the order *Methanosphaera spp.* use methanol for CH_4_ production [13].

Because neither of these microbes are abundant in the rumen [17], the contribution of these substrates to total CH_4_ production is expected to be lower [15]. Consequently, the most favourable CH_4_ production pathway in ruminants is the product of H_2_ oxidation using CO_2_ as an external electron acceptor [9].

### Methane mitigation strategies

Methane is expected to contribute approximately 18% of the total expected global warming within the next 50 years [18], of which the contribution of livestock to the total global emission is approximately 9% [3]. Domestic animals account approximately 94% of the total global emissions of animals [18]. Although emissions have decreased per unit of animal product, the total emission has increased from a vast animal population around the globe [4]. By 2050, the total CH_4_ emission from ruminant livestock is expected to increase significantly due to the growing demand of milk and meat for a rapidly growing world population [5]. Therefore, it is of utmost importance to mitigate CH_4_ emission from the livestock industry. There are several strategies for CH_4_ mitigation from ruminants that have recently been reviewed [19–21].

### Dietary manipulation

**Fig. 2:**
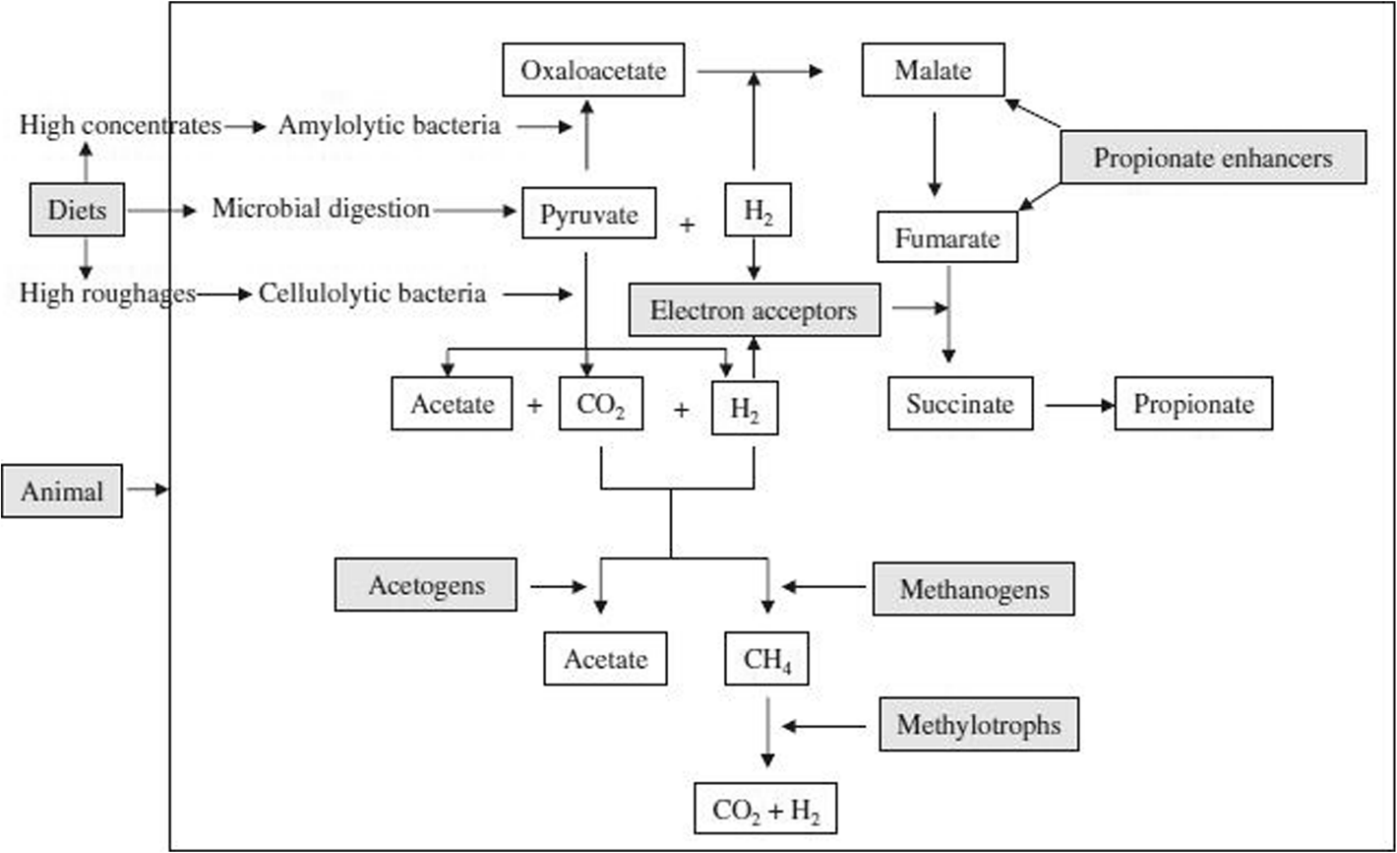
Target points (marked grey) at which dietary manipulation alters the fermentation pathway to reduce CH_4_ in the rumen [62]

Among the nutritional strategies of CH_4_ mitigation, dietary manipulation is a simplistic and pragmatic approach that can ensure better animal productivity as well as a lower CH_4_ emission. The schematic diagram of dietary manipulation, which alters the pathway of fermentation to reduce CH_4_, is summarised in Fig. 2.

Dietary manipulation can reduce CH_4_ emission up to 40% depending the degree of change and the nature of the intervention [22]. Another study also indicated that CH_4_ emissions can possibly be reduced up to 75% through better nutrition [23]. However, dietary manipulation is the most commonly practiced approach. Dietary strategies can be divided into two main categories: i) improving the forage quality and changing the proportion of the diet and ii) dietary supplementation of feed additives that either directly inhibit methanogens or altering the metabolic pathways leading to a reduction of the substrate for methanogenesis.

### Forage

Forage quality has influences CH_4_ production in the rumen [24]. High-quality forage, e.g., young plants, can reduce CH_4_ production by altering the fermentation pathway because this forage contains higher amounts of easily fermentable carbohydrates and less NDF, leading to a higher digestibility and passage rate [25]. In contrast, more mature forage induces a higher CH_4_ yield mainly due to an increased C:N ratio, which decreases the digestibility [18]. Different types of forage can also affect CH_4_ emission due to the differences in their chemical composition [22]. However, Hammond, Burke [26] found an inconsistent effect of the chemical composition of white clover and ryegrass on CH_4_ production. Legume forage has a lower CH_4_ yield, which is explained by the presence of condensed tannins, a low fibre content, a high dry matter intake and a fast passage rate [19]. Generally, C4 grasses yield more CH_4_ than the C3 plants [27]. Forage processing and preservation also affect CH_4_ emission [21]. For instance chopping or pelleting forages can reduce the CH_4_ emission per kg of DMI, as smaller particles require less degradation in the rumen [28]. Methanogenesis tends to be lower in the ensiled forages [28], presumably because the ensiled forages are already partially fermented during the ensiling process. Feeding improves the forage quality by feeding young forage with a lower fibre content and a higher soluble carbohydrate content; supplementing a small amount of grain with forage is a promising mitigation approach.

### Replacement of grass silage by maize silage

Grass silage is usually harvested at a later stage of maturity, resulting in a lower content of digestible organic matter, lower sugar and nitrogen contents and a fraction of lactate as a result of the ensiling process [29]. Consequently, the CH_4_ emission from animals that are fed grass silage is likely to be higher. In contrast, maize silage or other whole-crop small-grain silage typically provides higher contents of dry matter with readily digestible carbohydrates, e.g., starch, increasing the DMI and animal performance [19] and ultimately resulting in a lower CH_4_ yield from animals. There are three possible ways by which maize silage or whole-crop silage can reduce CH_4_ production in the rumen. First, the higher starch content favours propionate production rather than acetate. Second, the increased total DMI and passage rate reduce the ruminal residence time, thereby reducing ruminal fermentation and promoting post-ruminal digestion. Third, replacing grass silage with maize silage improves animal performance, resulting in fewer CH_4_ emissions per unit of animal product [30]. Several recent studies have indicated the positive effects of replacing grass silage with maize silage. Hassanat, Gervais [31] reported lower CH_4_ emission when alfalfa silage is replaced by 100% corn silage. Maize silage that is harvested during the later stage of maturity has also claimed to reduce CH_4_ [29].

### Concentrates

High-producing dairy cows have a higher requirement that exceeds their capacity to ingest nutrients from forage only. Therefore, forages must be supplemented with concentrates with a higher density of nutrients and less fibre. Due to less cell walls and readily fermentable carbohydrates (starch and sugar), concentrates favour propionic acid production, decreasing CH_4_ emission [21]. The CH_4_ reduction effect of concentrates can be described in two ways as below.

### Proportion of concentrate

The increased dietary level of concentrate reduces CH_4_ production as the energy proportion is mostly utilised by the animal products, such as milk and meat [21]. This effect is independent of genetic merit [32]. Decreased CH_4_ emission was observed at 80 and 90% concentrate supplementation, whereas no effect was found at 35 or 60% concentrate supplementation [33]. Most energy-rich concentrates are associated with increased DMI, rate of rumen fermentation and feed-turnover rate, causing a greater change in the rumen environment and microbial composition [21]. An extremely low CH_4_ loss of 2–3% of the gross energy intake was reported for feedlot cattle that were fed diet a 90% concentrate [34]. However, high-concentrate diets are low in structural fibre and in the long term disturb rumen function by leading to sub-acute or acute acidosis; therefore, these diets are not sustainable for ruminant production. Feeding concentrate with a suitable F:C ration would obviously be effective in methane mitigation as well as animal productivity.

### Concentrate composition

Concentrates that are composed of different ingredients have variable carbohydrate compositions, ranging from structural (cellulose and hemicellulose) to non-structural (starch and sugar) carbohydrates. The degradable rate of both of these types of carbohydrates also varies widely according to the volatile fatty acid profile and CH_4_ loss. In beef cattle [34], the digestion of the cell wall leads to a higher acetate: propionate ratio and CH_4_ loss compared to other carbohydrate fraction; within non-structural components, sugar is more methanogenic than starch. All of the carbohydrate fractions contribute to CH_4_ loss, of which the least contribution is that from starch, probably due to the maintenance of a propionate-dominating VFA profile [29]. Feeding more starch to ruminants reduces enteric CH_4_ energy losses compared to feeding a forage diet [35]. Starch fermentation promotes propionate production in the rumen by creating an alternative H_2_ sink [36], a lower rumen pH, inhibiting the growth of methanogens [37], decreasing the rumen protozoan numbers and limiting the interspecies H_2_ transfer between methanogens and protozoa [38]. In addition, feeding starch, which can escape rumen fermentation, could potentially supply energy to the host animals while avoiding methanogenesis in the rumen. Up to 30% of the starch from corn can escape rumen fermentation and be digested in the small intestine [39]. However, the bypass starch has limited digestibility (up to 60%) in the small intestine [40]. Very limited results are available on the effects of bypass starch on methane mitigation. Further investigation is required for detailed information.

In contrast, sugar as a water-soluble carbohydrate is rapidly and completely degradable in the rumen, enhancing butyrate production at the expense of propionate, thereby making sugar concentrates more methanogenic than starch [41]. Sugars enhance butyrate production at a higher H_2_ partial pressure and higher rumen pH, as confirmed by Hindrichsen and Kreuzer [42], who reported a 40% higher CH_4_ production with sucrose at a high pH compared to starch, while the opposite result was observed at a low pH with a significantly lower pH for sucrose.

### Fat supplementation

The addition of fat to the diet has traditionally been used to increase the dietary energy content to meet the energy demand of high-producing dairy cows. More recently, fat has been used for CH_4_ mitigation. If the energy supplementation in a ruminant’s diet is changed from carbohydrate to fat, then less fermentation and CH_4_ production will occur. The CH_4_-suppressing mechanism of fat is induced by reducing organic matter fermentation, fibre digestibility and consequently the methanogenic pathway and by the direct inhibition of methanogens in the rumen via the hydrogenation of unsaturated fatty acids [34]. The greatest reduction comes from the unsaturated fatty acids, which act as an H_2_ sink in the rumen through dehydrogenation [43], although other studies have reported that hydrogenation contributes only 1% of the H_2_ in the rumen [44]. Among fatty acids, the medium-chain C_8_:C_14_ from coconut or palm oil is the most effective in CH_4_ mitigation. Furthermore, fats are not metabolised in the rumen [45] and therefore do not contribute to methanogenesis [34]. Grainger and Beauchemin [46] also reported that fat supplementation often reduces carbohydrate fermentation due to the toxic effects of fat on cellulolytic bacteria and protozoa, while starch fermentation remains unaffected. Consequently, fat depresses CH_4_ emission [47]. However, fat supplementation to the ruminant diet is a persistent mitigation strategy [46].

### Organic acids

The addition of organic acids, the intermediates of carbohydrate degradation, to the rumen has been suggested as potential feed additives for CH_4_ mitigation. Organic acids probably stimulate propionic acid production in the rumen by acting as an H_2_ sink, thereby reducing the amount of CH_4_ [48]. Newbold, Lopez [49] tested 15 propionate precursors in vitro and concluded that the structure appears to be more effective as an H_2_ sink that can reduce CH_4_ up to 17%. Fumarate and acrylate produce the most consistent reductions in CH_4_ formation in batch cultures, while fumarate is more effective than acrylate in artificial rumens [50]. Furthermore, fumarate (3.5 g/L) reduces the CH_4_ output by 38% in continuous fermenters using forage as a substrate [51]. However, a meta-analysis [52] reported a lower CH_4_ reduction effect in a continuous batch culture. Including multiple forms of propionate precursors in the diet yielded an additive inhibition of CH_4_ emissions as the reductive pathways differ among organic acid sources [50]. In contrast, an in vivo study with growing beef cattle reported a potential beneficial change in rumen fermentation by fumarate, although CH_4_ reduction was unaffected [53]. Organic acid supplementation has mostly been tested for CH_4_ production in vitro, producing inconsistent results. Therefore, there is the potential to invest more research in farm animals.

### Essential oils

Essential oils are plant secondary metabolites, volatile components [29] and aromatic lipophilic compounds [54] with very strong antimicrobial properties [55], which inhibit the growth and survival of most of microorganisms in rumen [56]. The mode of action varies in individual essential oils [57]. However, all essential oils contain chemical constituents and functional groups, such as terpenoids, phenolic and phenols, which have strong antimicrobial properties. Because of their lipophilic nature, essential oils have a high affinity for microbial cell membranes, and functional groups interact with the microbial cell membrane [58]. Methanogenesis decreases with the application of essential oil, especially by reducing microbial populations. However, no effect has been observed so far on the major aspects of rumen fermentation [59]. Limited studies have investigated the effect on CH_4_ reduction in vivo. However, methanogenesis is inhibited by altering protein degradation and amino acid determination [59]. Further research needs to investigate the potential use of essential oils in mainstream livestock farming.

### Ionophores

Antibiotics, such as monensin, are antimicrobial compounds that are typically used in beef and dairy cattle production to modulate feed intake and improve feed efficiency and animal productivity [60]. Monensin increases the acetate: propionate ratio in rumen fermentation by increasing reducing equivalents that help to form propionate [19]. Monensin may also decrease ruminal protozoa. This antibiotic is typically added to the diet as premix or via a slow-releasing capsule and has an anti-methanogenic effect [19]. Ionophores do not alter the diversity of methanogens [61] but change the bacterial population from Gram-positive to Gram-negative with a consequent change in the fermentation from acetate to propionate, thereby reducing CH_4_ [62]. A high dose of monensin reduces CH_4_ production (g/d) by 4–10% in dairy and beef cattle [63, 64]. Furthermore, Guan, Wittenberg [65] reported a 30% CH_4_ reduction in beef cattle that were fed monensin (33 mg/kg), which was related to the number of ciliated protozoa. The inhibitory effects of ionophores on CH_4_ production may not persist over time, and microorganisms adapt to ionophores [19, 34, 65]. However, the possible transient effect of ionophores and increasing public pressure to reduce the use of antimicrobial feed additives in agricultural production will obviously limit the scope for a long-term solution to CH_4_ mitigation [19].

### Probiotics

The use of probiotics for CH_4_ mitigation has recently been described [66]; [43]. The specific CH_4_ reduction potential of probiotics has not been well documented due to the unsuccessful introduction of acetogens to the rumen as competitors of methanogens [67]. Probiotics, such as lactic acid producers (*Lactobacillus plantarum, L. casei, L. acidophilus and Enterococcus faecium*), acetate and propionate producers (*Selenomonas ruminantium and Megasphaera elsdenii*) and yeast (*Saccharomyces cerevisiae and Aspergillus oryzae*) are widely used for the health of both human and animals [68]. Probiotics based on *Saccharomyces cerevisiae* are increasingly used in ruminant diets to improve rumen fermentation, dry matter intake and milk yield [19]. The underlying mechanism is probably the alteration of H_2_ production by the increased number of bacteria due to the partitioning of degraded carbohydrates between the microbial cells and fermented products [69]. Due to their modest price and wide use in ruminant production, the acceptance of CH_4_-reducing probiotics has a high probability in CH_4_ abatement. However, further research is needed to investigate the best possible products [19].

### Exogenous enzymes

Enzymes, such as cellulase and hemicellulase, are currently being used in ruminant diets. When properly formulated, enzymes can improve fibre digestibility and animal productivity [70]. Enzymes that improve fibre digestibility typically lower the acetate: propionate ratio in the rumen, ultimately reducing CH_4_ production [71]. Subsequently, in a recent review, Beauchemin, Kreuzer [19] suggested the possibility of developing a commercial enzyme additive to reduce CH_4_. However, searching for potential enzymes for methane abatement warrants future research.

### Alternative H_2_ sink

Alternative H_2_ sinks, for example, nitrate and sulphate, are used at lower concentrations in the basic diets of ruminants. As alternative electron acceptors, nitrate and sulphate have a greater reduction potential and are thermodynamically highly favourable for some rumen microbes [72]. Regarding methane mitigation, Leng [73] described the potential of nitrate supplementation in the ruminant diet. Furthermore, van Zijderveld, Gerrits [74] demonstrated that the reduction effect of nitrate and sulphate is electronically more favourable than is CH_4_ production, which can potentially change the competitiveness of H_2_ scavengers. In recent years, nitrate and sulphate have been increasingly tested for CH_4_ abatement. A 32% methane reduction was reported for nitrate, 16% for sulphate and 47% for a combination of nitrate and sulphate fed to lambs [74]. The same author in a subsequent study indicated an approximately 16% CH_4_ (g/d and g/kg DMI) reduction in dairy cows [75]. However, nitrate supplementation has not been established in many countries (e.g., in Denmark) due to toxic effects that could lead to animal death. One potential toxic effect occurs via the reduction of nitrate to nitrite, which causes methemoglobinemia, a condition in which blood haemoglobin cannot carry oxygen [74]. Because a lower amount of nitrate in the diet is safe for the animal [76], nitrate supplementation can be an effective CH_4_ mitigation measure. However, more research is needed to determine the inclusion levels for different ruminant species.

### Plant secondary metabolites

The potential effect of plant secondary metabolites (PSM) in CH_4_ reduction has been recently recognised [19]. The CH_4_-suppressing effect of PSM is mainly associated with antimicrobial properties that kill the bacteria [77], protozoa [78] and fungi [79] in the rumen. Plant secondary metabolites contain phenolic compounds the main active components that have antimicrobial activity [80]. Plants produce a variety of secondary compounds, among which condensed tannins [81] and saponins [82] have received much attention.

### Condensed tannins

An interesting development in CH_4_ mitigation research is the development of forages with higher levels of tannins, such as clover and other legumes, including trefoil, vetch, sulla and chicory [29]. The anti-methanogenic activity of tannins has recently been investigated in vitro and in vivo [83]. The CH_4_-suppressing mechanism of tannins has not been described clearly; however, this mechanism may inhibit ruminal microorganisms [77]. Tannins may inhibit, through bactericidal or bacteriostatic activities, the growth or activity of rumen methanogens and protozoa [84]. Methane production was reduced (up to 55%) when ruminants were fed tannin-rich forages, such as lucerne, sulla, red clover, chicory and lotus [81]. Although tannins appear promising for CH_4_ mitigation, these impede forage digestibility and animal productivity when fed at a higher concentration, limiting their future wide-scale use in CH_4_ abatement [19]. However, more research may identify the balance between CH_4_ reduction and possible anti-nutritional side effects as associated with tannin supplementation.

### Saponins

Saponins are naturally occurring surface-active glycosides that are found in a wide variety of cultivated and wild plant species that reduce CH_4_ production in the rumen [29, 79]. Saponins have a potent antiprotozoal activity by forming complex sterols in protozoan cell membranes [83] and, to some extent, exhibit bacteriolytic activity in the rumen [66]. Saponins are antiprotozoal at lower concentrations [85], whereas higher concentrations can suppress methanogens [77]. Saponins inhibit ruminal bacterial and fungal species [79] and limit the H_2_ availability for methanogenesis in the rumen, thereby reducing CH_4_ production [77]. Methane reduction of up to 50% has been reported with the addition of saponins [86]. However, a wider range of CH_4_ reduction (14–96% depending on the plant and the solvent that was used for extraction) has been reported [62].

### Rumen manipulation

Manipulating the microbial diversity in the rumen through chemical means (e.g., halogenated compounds and chloroform) by introducing competitive or predatory microbes or through direct immunisation can reduce methanogenesis in ruminants [20]. A preliminary study suggested that vaccination against methanogens can reduce CH_4_ emission up to 8% [87]. However, the long-term effect of vaccination on CH_4_ reduction is still uncertain [88]. Furthermore, methanogen populations in the rumen are influenced by diet and geographic location (Wright et al., 2007); therefore, it is challenging to develop a broad-spectrum vaccine against all methanogens. Instead, the development of a vaccine against the cell-surface proteins of methanogens may improve the efficacy of vaccination for CH_4_ mitigation [50]. Biological control bacteriophages or bacteriocins could be effective in the direct inhibition of methanogens and in redirecting H_2_ to other reductive rumen microbes, such as propionate producers or acetogens [50]. However, most of these options are still conceptual, and significant research is required.

Halogenated compounds, such as bromochloromethane and chloroform, are potent inhibitors of CH_4_ production in ruminants. Methane reduction has been reported with bromochloromethane mainly due to the reduction of methanogen abundance [89]. An approximately 26% CH_4_ reduction was reported by McAllister and Newbold [50] through the chemical inhibition of protozoa because the methanogens are often attached to the surface or endosymbionts within ciliated protozoa [50].

Defaunation also reduces CH_4_ emission. Two major advantages of defaunation are that it increases nutrient utilisation by animals and limits H_2_ transfer between protozoa and methanogens. The methanogens that are attached to ciliated protozoa contribute approximately 9–37% of the methanogenesis in the rumen [38]. Protozoa-free lambs and sheep exhibits 26 and 20% CH_4_ reduction, respectively [50]. The elimination of the protozoan population in CH_4_ mitigation is interesting, but the absence of protozoa in the rumen can hinder digestibility and animal performance.

Reductive acetogenesis, in which H_2_ and CO_2_ form acetate rather than CH_4_ as a source of energy, has been suggested as an alternative to methanogenesis [90]. The production of acetate instead of CH_4_ can increase the energy supply to the animals. Joblin [90] suggested that if the CH_4_ emissions in ruminant were fully replaced by acetate, this could represent an energetic gain of 4–15%. However, acetogenesis in CH_4_ reduction has not been successful due to the failure in acetogens competing for H_2_ in the rumen. Research in acetogenesis as a CH_4_ mitigation measure is still in the initial phase and warrant more research.

### Animal manipulation

Several options, such as culling low-producing animals, increasing animal productivity and breeding animals with lower CH_4_, have been suggested for CH_4_ mitigation through animal manipulation. Methane emission is directly proportional to the number of animals in a herd. The replacement of non-productive and low-producing animals would cut the total CH_4_ budget from the herd. Maintaining high-producing animals will increase the total production, but the CH_4_ emission per unit of animal product will decrease [62, 91]. Therefore, proper nutrition management to improve productivity is an option to reduce the CH_4_ emission per unit of animal product.

Several studies have demonstrated a substantial variation in CH_4_ production in sheep and cows [92–94], which may be linked to phenotypic traits and heritability. This animal variation in CH_4_ production suggests a possibility of breeding animals with low CH_4_ emission. However, Eckard, Grainger [20] suggested that breeding for reduce CH_4_ production is unlikely to be compatible with other breeding objectives.

## Conclusions

A Number of methane mitigation options are available and currently in practice. No single option appears to provide a simple and enduring solution. Selection and breeding of low methane emitter animals is one of the solutions which requires longer time frame. Use of chemicals, ionophors, plant secondary metabolites or such application attributes transitory effects on methane reduction. However, overall dietary manipulation by selecting and utilizing high quality forages, strategic supplementation of forages, changing concentrate proportion with special emphasis on changing carbohydrate composition should be considered as an immediate and sustainable methane mitigation approach of enteric CH_4_ emitted from ruminant livestock. Feeding a diet with more starch and less fibres not only produce less methane per kg feed DM but also form a basis for higher feed intake and higher production per animal and hence will be the most efficient way to reduce the methane production per kg of meat or milk produced.

## Abbreviations

GHG: Greenhouse gas
CO_2_: Carbon dioxide
CH_4_: Methane
N_2_O: Nitrous oxide
GWP: Global warming potential
FPCM: Fat and protein corrected milk
DMI: Dry matter Intake
H_2_: Hydrogen
VFA: Volatile fatty acid
